# Gut Microbiome Diversity and Composition in Captive Siberian Tigers (*Panthera tigris altaica*): The Influence of Diet, Health Status, and Captivity on Microbial Communities

**DOI:** 10.3390/microorganisms12112165

**Published:** 2024-10-27

**Authors:** You-Jeong Lee, Saebom Lee, Beoul Kim, Dongmi Kwak, Taehwan Kim, Min-Goo Seo

**Affiliations:** 1 College of Veterinary Medicine & Institute for Veterinary Biomedical Science, Kyungpook National University, 80 Daehak-ro, Buk-gu, Daegu 41566, Republic of Korea; wowgirlsgood@naver.com (Y.-J.L.); kbjjhnm@naver.com (B.K.); dmkwak@knu.ac.kr (D.K.); 2 Baekdudaegan National Arboretum, Korean Tiger Conservation Center, 1501 Chunyang-ro, Chunyang-myeon, Bonghwa 36209, Republic of Korea; gltp43@koagi.or.kr

**Keywords:** gut microbiome, Siberian tiger, alpha diversity, beta diversity, taxonomy profiling, conservation, captivity

## Abstract

The gut microbiome is essential for the health of carnivorous mammals, including the endangered Siberian tiger (*Panthera tigris altaica*). However, limited research exists on the gut microbiome of captive Siberian tigers, especially regarding how diet and health status influence microbial diversity. This study addresses this gap by investigating the gut microbiome diversity and composition of six captive-born Siberian tigers housed at the Baekdudaegan National Arboretum in South Korea, using 16S rRNA gene sequencing. The study aimed to examine how diet and health status influence microbial communities, providing baseline data for managing captive tigers. Alpha diversity analysis revealed significant variation in microbial richness and evenness, with Tigers 2 and 6 exhibiting the highest microbial diversity and Tiger 3 the lowest, likely due to its surgical history and limited diet. Beta diversity analysis showed distinct microbial community structures influenced by diet and health. Taxonomic profiling identified *Firmicutes* and *Bacteroidota* as the dominant phyla, with *Clostridium* sensu stricto more prevalent in healthier tigers, while *Escherichia*-*Shigella* and *Proteobacteria* were abundant in tigers with lower diversity, suggesting dysbiosis. Comparisons with other tiger species confirm that diet, health, and captivity significantly shape the gut microbiome. These findings highlight the need for personalized health management in captive environments.

## 1. Introduction

The Siberian tiger (*Panthera tigris altaica*), commonly called the Amur tiger, ranks among the most critically endangered tiger subspecies. Its survival faces significant threats from habitat loss, poaching, and the depletion of its prey species [1]. Conservation efforts traditionally focus on habitat preservation and anti-poaching measures. However, attention has recently shifted towards understanding the health and physiology of captive populations, which play a vital role in species survival programs. One emerging area of interest is the gut microbiome—the diverse community of microorganisms in the digestive tract. This microbiome influences various aspects of animal health, including immune function, nutrient absorption, and overall physiological homeostasis [2].

In carnivorous mammals, such as tigers, the gut microbiome is primarily composed of bacterial phyla like *Firmicutes*, *Bacteroidota*, *Actinobacteriota*, and *Proteobacteria* [3]. These microorganisms aid in digesting complex carbohydrates and proteins, producing metabolites such as short-chain fatty acids (SCFAs), which are crucial in energy metabolism and gut health [4]. However, several factors influence the composition of the gut microbiome, including diet, health status, and environmental conditions, such as those experienced in captivity [5]. Research shows that captivity often alters microbial diversity and composition, potentially affecting animal health, particularly in long-term captive animals [6].

Growing evidence shows distinct gut microbiome compositions in wild and captive tigers, as demonstrated by studies on various tiger subspecies. For example, in wild Malayan tigers (*Panthera tigris jacksoni*), habitat and diet significantly shape microbial diversity, with distinct patterns observed among tigers from different environments [7]. In captive Malayan tigers, fasting and dietary changes influence gut microbiome diversity, particularly increasing the abundance of *Proteobacteria*, a pattern also observed in other captive environments [8]. Similarly, studies on Amur tigers (*Panthera tigris altaica*) show variations in microbial diversity based on health status and captivity conditions [3]. In addition, research on South China tigers shows that developmental stages and environmental factors dynamically shape gut microbiome composition, underscoring the critical role of diet and health in managing captive populations [9].

Despite the significance of understanding these microbial communities, research on the gut microbiome of Siberian tigers, especially in captive settings, remains limited, particularly in South Korea. Most existing studies focus on related tiger species or wild populations, leaving gaps in our understanding of how captivity and diet affect gut microbiota in this subspecies [3]. Understanding these microbial communities is essential for improving the health and welfare of captive tigers and informing conservation strategies, as optimal gut health influences the overall fitness and survival of animals reintroduced into the wild [10].

Therefore, this study aims to investigate the diversity and composition of the gut microbiome in six captive-born Siberian tigers at the Baekdudaegan National Arboretum (Korean Tiger Conservation Center) in South Korea. We hypothesize that diet and health conditions could significantly alter microbial diversity, influencing the management and conservation of captive tiger populations. Our findings could provide baseline data for captive Siberian tigers and emphasize the importance of personalized management to promote a healthy gut microbiome.

## 2. Materials and Methods

### 2.1. Ethical Approval

All experimental procedures were approved by the Institutional Animal Care and Use Committee at Kyungpook National University (KNU 2024-0026, approval date: 22 January 2024). Fecal samples were collected noninvasively to ensure the animals were not harmed. The collection process was conducted with minimal disturbance, adhering to ethical standards for wildlife research.

### 2.2. Sample Collection and Health Status

Fecal samples were collected in November 2022 from each of the six captive-born Siberian tigers (Tigers 1–6) housed at the Baekdudaegan National Arboretum in Bonghwa, South Korea. Table 1 provides detailed information on the tigers, including their age, diet, weight, and sex. At the time of fecal sample collection, all six tigers were clinically healthy, although some had previous health conditions. Tiger 1 experienced intestinal inflammation in June 2022, while Tiger 3 previously underwent a vasectomy. Tiger 5—the oldest of the group—exhibited age-related symptoms such as tremors and tooth loss, while Tiger 6 was in estrus. These factors were considered when interpreting the microbiome results. The samples were aseptically collected immediately post-defecation and stored in sterile containers. Within 24 h, the samples were transported on ice to the laboratory and stored at −80 °C until DNA extraction.

### 2.3. DNA Extraction

Approximately 200 mg of each fecal sample was used for DNA extraction with the QIAGEN PowerSoil DNA Kit (Qiagen, Hilden, Germany) following the protocol of the manufacturer. DNA concentration and purity were assessed using a Nanodrop spectrophotometer and Qubit fluorometer (Thermo Fisher Scientific, Waltham, MA, USA). DNA extraction yielded approximately 900 ng per sample, and DNA integrity was further validated using a Bioanalyzer (Agilent RNA 6000 Pico Kit, Agilent, Santa Clara, CA, USA).

### 2.4. 16S rRNA Gene Amplification

The hypervariable V3–V4 region of the 16S rRNA gene was amplified using primers 16S-F (5′-CCTACGGGNGGCWGCAG-3′) and 16S-R (5′-GACTACHVGGGTATCTAATCC-3′). These primers targeted conserved regions of the gene while capturing the variability of the intervening sequences. These primers were selected for their effectiveness in previous studies on microbial diversity [3].

### 2.5. Library Preparation and Sequencing

PCR products were purified using AMPure XP beads (Beckman Coulter, Brea, CA, USA) to eliminate residual primers and contaminants. Purified amplicons were quantified using a Qubit fluorometer and pooled in equimolar concentrations to construct the sequencing library. The library was prepared using the Nextera XT DNA Library Preparation Kit (Illumina, San Diego, CA, USA) according to the protocol of the manufacturer. Sequencing was performed on an Illumina MiSeq platform using 2 × 300 bp paired-end sequencing. Quality control and cluster generation were automated, ensuring the equal distribution of sequence reads among the samples.

### 2.6. Quality Control and Preprocessing

Raw sequencing reads were evaluated using FastQC (v0.10.1, Babraham Institute, Cambridge, UK) for base quality, GC content, and adapter sequences. Reads with a Phred score < 20 were trimmed using Cutadapt (v3.2, Marcel Martin, Freiburg, Germany), and residual adapter sequences were eliminated. Low-quality reads with a Phred score < 20, phiX reads, chimeric reads, and duplicates were filtered out using the DADA2 pipeline (v1.20.0, R Development Core Team, Vienna, Austria) [11], which was also used to denoise the data and produce amplicon sequence variants (ASVs). The parameters for the DADA2 pipeline were set as follows: maxEE = 2, truncQ = 2, truncLen = c(250,200), and maxN = 0. The output consisted of ASVs, which are preferred for improved taxonomic resolution over traditional OTUs. Quality-filtered sequences were demultiplexed, followed by processing using the QIIME2 pipeline (v2021.11, Caporaso Lab, Flagstaff, AZ, USA) for taxonomic classification [12]. ASVs were classified according to the SILVA reference database (v138.99, Max Planck Institute, Bremen, Germany). Operational taxonomic units were clustered at a 97% similarity threshold using the Greengenes reference database (v13_5, Lawrence Berkeley National Laboratory, Berkeley, CA, USA) [13].

### 2.7. Alpha Diversity Analysis

Alpha diversity was assessed to measure species richness and evenness within each sample. Several indices were calculated, including Chao1 (species richness), Shannon Entropy (richness and evenness), Simpson’s Diversity Index (evenness), Pielou’s Evenness, and Faith’s Phylogenetic Diversity (phylogenetic relationships between taxa). The Wilcoxon Rank-Sum Test was used to assess statistical differences between the samples. Multiple indices were employed to ensure robustness and cross-validation of the findings despite some overlap in their measurements of richness and evenness.

### 2.8. Beta Diversity Analysis

Beta diversity analysis was employed to assess differences in the microbial community composition among the six Siberian tigers. We used multiple beta diversity metrics to capture various aspects of microbial community structure and dissimilarities. The Bray–Curtis dissimilarity index, a quantitative metric, accounts for the abundance of shared taxa between samples and emphasizes compositional differences by considering the presence and relative abundance of taxa. The Jaccard distance metric focuses on the presence or absence of taxa, disregarding abundance and measuring the similarity in community membership by determining whether taxa were shared or unique between samples. Besides the abundance-based and presence/absence-based metrics, phylogeny-based UniFrac metrics were employed. The unweighted UniFrac metric is employed to measure the dissimilarity between microbial communities based on the presence or absence of phylogenetically distinct taxa, while the weighted UniFrac metric incorporates the relative abundances of taxa, offering a more refined view of dissimilarity by weighting taxa-based on their proportions.

Principal Coordinate Analysis (PCoA) was applied to the Bray–Curtis dissimilarity data to visualize the differences revealed by these metrics and illustrate the separation between microbial communities based on their compositional differences. Non-metric multidimensional scaling (NMDS) was employed for Jaccard distance, unweighted UniFrac, and weighted UniFrac metrics. It offers complementary views of the microbial community differences.

To statistically assess the differences in beta diversity between tiger groups, Permutational Multivariate Analysis of Variance (PERMANOVA) was conducted using the adonis2 function within the vegan R package (v2.5–7, R Core Team, Vienna, Austria). This analysis was conducted using 999 permutations to evaluate the significance of group differences [14].

Three different grouping configurations were employed in the beta diversity analysis to explore the influence of factors such as diet and health conditions on microbial community composition. In Analysis 1, Tigers 1, 2, and 3 were compared with Tigers 4, 5, and 6. In analysis 2, Tigers 1, 2, and 4 were compared with Tigers 3, 5, and 6. Finally, in Analysis 3, Tigers 2, 4, and 5 were compared with Tigers 1, 3, and 6. These grouping strategies enabled the assessment of how diet and health status influenced microbial community composition. We captured taxonomic and phylogenetic differences between the groups using multiple beta diversity metrics, which provides a comprehensive understanding of the factors driving microbial diversity in captive Siberian tigers.

### 2.9. Taxonomy Profiling

The taxonomic composition of the microbial communities was analyzed at the phylum, class, order, family, and genus levels using the SILVA database (v138.99, Max Planck Institute, Bremen, Germany) for taxonomic classification. Differential taxa abundance was evaluated using the Analysis of Composition of Microbiomes with Bias Correction to adjust for compositional bias [15].

## 3. Results

### 3.1. Alpha Diversity Outcomes and Interpretations

**Figure 1:**
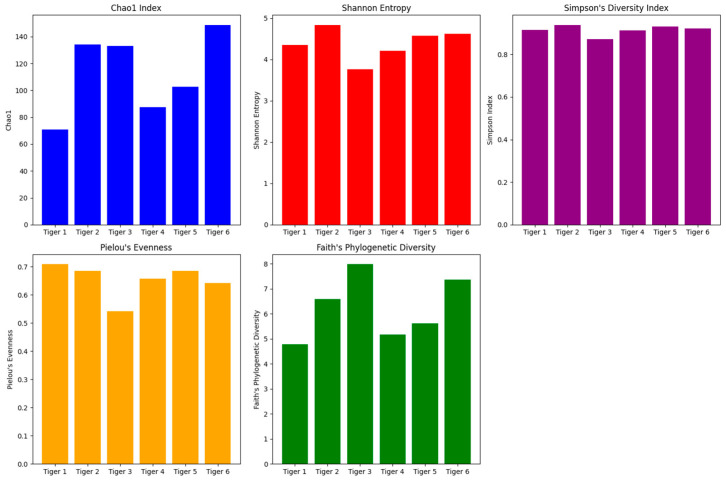
Bar plots illustrating the alpha diversity indices (Chao1, Shannon Entropy, Simpson’s Diversity Index, Pielou’s Evenness, and Faith’s Phylogenetic Diversity) for the gut microbiome of six Siberian tigers.

Alpha diversity indices provide insights into species richness, evenness, and phylogenetic diversity within the gut microbiomes of the six Siberian tigers. Figure 1 depicts the visualized results.

Tigers 2 and 6 exhibited the highest species richness and phylogenetic diversity, as evidenced by their elevated Chao1 and Faith’s Phylogenetic Diversity scores. In contrast, Tiger 3 demonstrated high species richness, as indicated by its Chao1 and Faith’s Phylogenetic Diversity scores. However, it exhibited the lowest Shannon Entropy and Simpson’s Diversity Index, indicating reduced microbial evenness. Statistical analysis using the Wilcoxon Rank-Sum Test (Supplementary Figure S1) revealed significant differences in alpha diversity metrics, particularly between Tigers 6 and 3 (*p* < 0.05).

### 3.2. Beta Diversity Outcomes and Interpretations

Beta diversity was analyzed to compare the microbial community composition among the six tigers. This analysis was conducted using Bray–Curtis dissimilarity, Jaccard distance, unweighted UniFrac, and weighted UniFrac metrics.

**Figure 2:**
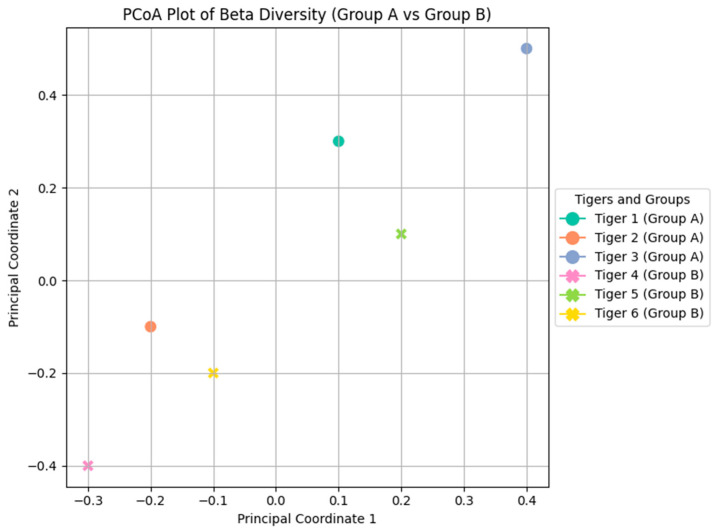
PCoA plot illustrating the beta diversity analysis comparing Group A (Tigers 1, 2, and 3) and Group B (Tigers 4, 5, and 6) based on Bray–Curtis dissimilarity.

The PCoA plot (Figure 2) depicts distinct clustering patterns based on Bray–Curtis dissimilarity, indicating a clear separation between Groups A (Tigers 1, 2, and 3) and B (Tigers 4, 5, and 6). This clustering suggests that the microbial communities in Group A tigers are more similar to one another than those in Group B. Tigers 3 and 6 exhibited the most dissimilar microbiota, potentially influenced by their distinct health conditions and dietary differences. The NMDS plot (Supplementary Figure S2) provides a complementary visualization that confirms the separation between the two groups using the same dissimilarity metric. PERMANOVA analysis confirmed statistically significant differences in microbial composition between the groups, with a *p*-value < 0.05 (Table 2).

The Jaccard distance metric, which focuses on the presence or absence of taxa, also revealed a clear separation between Groups A and B. In the NMDS plot (Supplementary Figure S3), Tiger 3 exhibited the greatest divergence in microbial community membership among all tigers, further supporting the microbial dysbiosis hypothesis. This divergence suggests that the microbial composition in Tiger 3 is distinct from that of the other individuals, possibly linked to health or environmental factors. PERMANOVA analysis revealed statistically significant differences between the groups (*p* < 0.05).

The phylogeny-based UniFrac metrics provided further insights into the microbial community composition. The unweighted UniFrac plot (Supplementary Figure S4) highlighted the presence or absence of phylogenetically distinct taxa, with Tiger 3 identified as an outlier. This indicates that Tiger 3 harbored unique microbial lineages not present in other tigers. The weighted UniFrac plot, which incorporates the relative abundances of taxa, showed that Tigers 4 and 5 had more similar microbial communities than the other tigers, potentially driven by shared dietary patterns. Both metrics revealed statistically significant differences between the groups based on PERMANOVA analysis (*p* < 0.05).

### 3.3. Taxonomic Composition

**Figure 3:**
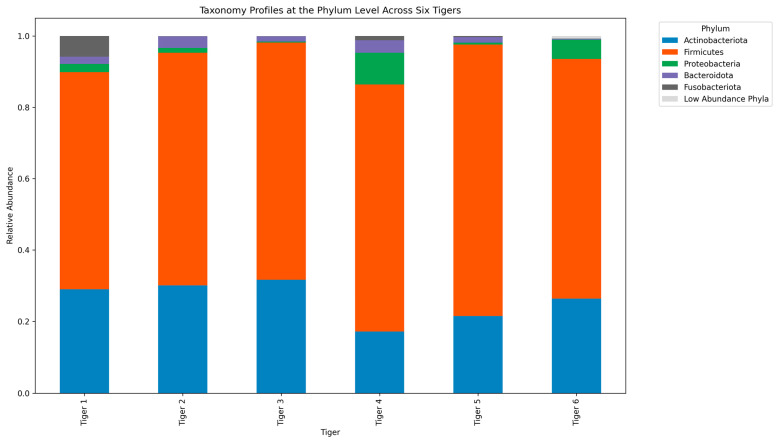
Stacked bar plot depicting the relative abundance of microbial communities at the phylum level across the six Siberian tigers.

**Figure 4:**
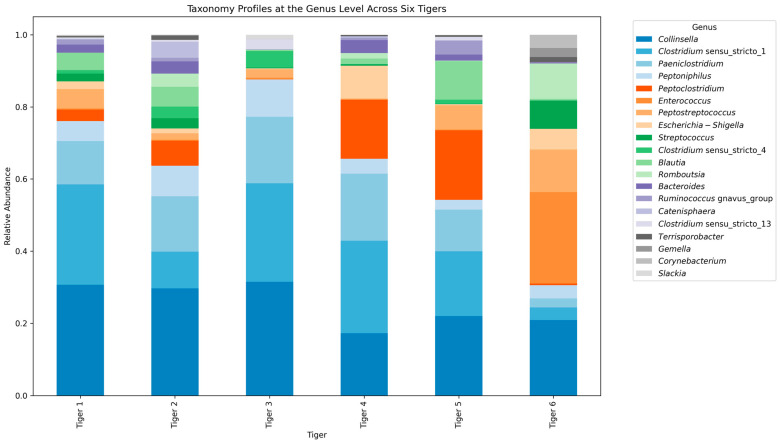
Stacked bar plot illustrating the relative abundance of microbial communities at the genus level among the six Siberian tigers.

We analyzed the taxonomic composition of the gut microbiome in the six Siberian tigers at the phylum, class, order, family, and genus levels. Figure 3 and Figure 4 depict the visualized results, while Supplementary Figures S5–S7 provides the supplementary taxonomic details. Table 3 shows the dominant taxa at each taxonomic level.

At the phylum level, the dominant taxa across all tigers were *Firmicutes* (52.8%) and *Bacteroidota* (8.3%) (Figure 3). *Proteobacteria* levels varied, with Tiger 3 exhibiting the highest abundance. Unclassified reads at the phylum level ranged from 0% to 0.01%.

At the class level, *Clostridia* (38.4%) was the most prevalent class among all tigers, particularly in Tigers 2 and 6 (Supplementary Figure S5). Tiger 3 exhibited elevated *Gammaproteobacteria* levels, while unclassified reads at the class level ranged from 0% to 0.09%.

At the order level, *Clostridiales* (34.2%) was the dominant microbial order across all tigers (Supplementary Figure S6), while *Enterobacterales* levels were elevated in Tiger 3. Unclassified reads at the order level ranged from 0% to 0.12%.

At the family level, *Lachnospiraceae* (27.6%) was highly abundant in Tigers 2 and 6 (Supplementary Figure S7), while *Enterobacteriaceae* levels were elevated in Tiger 3. Unclassified reads at the family level ranged from 0% to 0.22%.

At the genus level, *Clostridium* sensu stricto (19.5%) was the most dominant genus across all tigers, particularly abundant in Tigers 2 and 6 (Figure 4), while *Escherichia*-*Shigella* was more prevalent in Tiger 3. Unclassified reads at the genus level ranged from 0% to 0.57%, with Tiger 1 exhibiting the highest percentage.

## 4. Discussion

This study provides valuable insights into the diversity and composition of the gut microbiome of six captive Siberian tigers, highlighting the correlation between diet, health status, and microbial community structure. The findings suggest that individual factors, such as diet and health, significantly influence microbial diversity in these endangered animals. To contextualize these findings, comparing them with other studies on tiger microbiomes, particularly those involving wild and captive populations, is essential.

The alpha diversity analysis revealed substantial variation among the tigers, with Tigers 2 and 6 showing the highest species richness and phylogenetic diversity, which could result from their varied diets and healthier conditions. In contrast, Tiger 3 exhibited the lowest Shannon entropy and Simpson’s diversity index, suggesting reduced microbial evenness. This low evenness may be associated with microbial dysbiosis, potentially linked to the health status and surgical history of Tiger 3, which may have disrupted the balance of its gut microbiota. Lower microbial evenness often indicates an imbalanced gut, which could increase susceptibility to health challenges, particularly in carnivorous mammals such as tigers [2].

Our findings align with those of previous studies on captive Malayan tigers (*Panthera tigris jacksoni*), which also show microbial imbalances during periods of dietary changes and health stress. For example, Khairulmunir et al. indicate that captive Malayan tigers undergoing fasting exhibit shifts in microbial diversity, with increased levels of *Proteobacteria* [8]. This finding is similar to our observation of elevated *Proteobacteria*, particularly *Escherichia*-*Shigella*, in Tiger 3. These findings reinforce the notion that health status and diet drive gut microbial changes in captive tigers; however, our findings should be interpreted cautiously owing to the small sample size.

Beta diversity analysis, visualized through PCoA and NMDS plots, revealed distinct microbial community structures between the tiger groups. Groups A (Tigers 1, 2, and 3) and B (Tigers 4, 5, and 6) exhibited significant differences in microbial composition, with Tiger 3 consistently diverging from others across all beta diversity metrics. This separation suggests that the health condition of Tiger 3, along with its diet, contributes to its unique microbial composition. PERMANOVA analysis revealed significant differences in microbial communities, confirming that diet and health conditions are key drivers of variations among these tigers [2]. These findings align with those of Gani et al. on wild Malayan tigers, which demonstrates that habitat and environmental factors, rather than health alone, significantly influence gut microbial diversity [7].

Although our study lacks wild controls, this comparison suggests that captivity itself contributes to reduced microbial diversity in Tiger 3, whose microbial community may be more susceptible to imbalances under these conditions. Without these comparisons, drawing definitive conclusions about whether the observed differences are driven by captivity or if they reflect natural variations among individuals is challenging.

At the taxonomic level, *Firmicutes* and *Bacteroidota* were the dominant phyla among all six tigers. *Firmicutes*, known for their role in fiber digestion and SCFA production, were more prevalent in Tigers 2 and 6. This higher abundance correlates with the overall increased microbial richness and dietary diversity [5]. These bacteria are crucial for the gut health of carnivorous mammals and are abundantly present in these tigers, suggesting a healthy gut microbiota capable of breaking down complex dietary fibers [3]. In contrast, Tiger 3 exhibited elevated levels of *Proteobacteria*, particularly *Escherichia*-*Shigella*, which is commonly linked to gut infections and dysbiosis. This supports the earlier observation that Tiger 3 may have experienced microbial imbalance owing to its health condition, as dysbiosis is often linked to gastrointestinal distress in wild and captive animals [4].

At the class level, *Clostridia* (38.4%) was dominant across all tigers, particularly in Tigers 2 and 6, where it plays a crucial role in SCFA production. In contrast, *Gammaproteobacteria*, a class of *Proteobacteria* that includes several opportunistic pathogens, was more prevalent in Tiger 3, indicating microbial dysbiosis. This class includes pathogenic bacteria such as *Escherichia*-*Shigella*, which are often linked to gastrointestinal disorders [7].

*Clostridiales* (34.2%) predominated at the order level, especially in Tigers 2 and 6, where it contributes to a healthier gut microbiota by facilitating carbohydrate fermentation and SCFA production [8]. In contrast, *Enterobacterales*—an order within *Gammaproteobacteria*—was prevalent in Tiger 3, further suggesting dysbiosis, with elevated levels of *Escherichia*-*Shigella* detected [9].

At the family level, *Lachnospiraceae* (27.6%) was highly prevalent in Tigers 2 and 6, which is associated with the fermentation of dietary fibers and SCFA production that promotes gut health [8]. However, *Enterobacteriaceae*, commonly linked to gastrointestinal disorders, showed higher abundance in Tiger 3, indicating a potential microbial imbalance in this tiger [9].

At the genus level, *Clostridium* sensu stricto and *Lactobacillus* were prevalent in Tigers 2 and 6, indicating a healthier gut microbiome composition. Both genera play a crucial role in maintaining gut health by producing SCFA and modulating the immune system [6]. In contrast, *Escherichia*-*Shigella*, often linked to gastrointestinal infections, was more abundant in Tiger 3, further supporting the hypothesis of microbial dysbiosis. These findings align with that of previous studies on tiger microbiomes, such as South China tigers, which show similar microbial shifts in response to dietary changes and health status across various developmental stages [9].

A significant observation in this study was the low relative abundance of unclassified reads, especially at lower taxonomic levels such as the genus. The use of high-quality reference databases, such as SILVA, along with stringent filtering criteria during bioinformatics processing, ensured accurate taxonomic assignment. The low proportion of unclassified organisms (ranging from 0% to 0.57%) indicates high confidence in the identified taxa. However, the minimal unclassified reads could represent novel or understudied microbial species that are not well-represented in current databases [16]. Future research should prioritize expanding microbial reference databases or utilizing metagenomic approaches to characterize unclassified taxa, potentially revealing crucial contributions to gut health [16].

Although this study yields valuable insights, its small sample size of six tigers limits the statistical power and generalizability of the results. The absence of wild controls further complicates our ability to draw definitive conclusions regarding the effect of captivity on the gut microbiome. Comparing our findings with those of wild populations, such as wild South China and wild Malayan tigers [7,9], indicates that natural variations may contribute to some differences observed in our study. Consequently, larger sample sizes and more comprehensive analyses, including comparisons with wild populations, are essential to distinguish between captivity-driven and naturally occurring individual variability in the gut microbiome composition.

Moreover, the lack of functional analyses in this study limits our understanding of the metabolic capabilities of the identified microbial taxa. Functional analyses, such as metagenomics or metabolomics, could provide valuable insights into the microbial functions supporting gut health and elucidating the physiological roles of specific taxa [14,17]. Additionally, longitudinal studies enable tracking changes in the gut microbiome over time, providing a better understanding of the long-term effects of diet and captivity.

In conclusion, this study shows the importance of personalized dietary and health management strategies for captive Siberian tigers. Diet and health conditions critically influence the gut microbiome, and maintaining a balanced and diverse gut microbiota is vital for the overall health and well-being of these endangered animals. Nonetheless, future studies should integrate larger sample sizes, functional analyses, and comparisons with wild populations to elucidate the effects of captivity on the gut microbiome of Siberian tigers [15,17].

## 5. Conclusions

This study provides valuable baseline data on the gut microbiome diversity and composition of six captive-born Siberian tigers at a conservation facility. Our findings indicate the significant effect of diet, health status, and captivity on the gut microbial communities of these endangered animals. The alpha diversity analysis revealed that Tigers 2 and 6, which have more varied diets and healthier conditions, exhibited higher microbial richness and phylogenetic diversity. In contrast, Tiger 3, with a history of surgery and health issues, displayed lower microbial evenness and signs of microbial dysbiosis, including elevated levels of *Proteobacteria* and *Escherichia*-*Shigella*.

Comparing our findings with those of previous studies on tiger microbiome, including those on captive Malayan, wild Malayan, captive Amur, and South China tigers, confirms that diet, health conditions, and captivity significantly influence gut microbial diversity across different tiger species. These comparisons reveal common microbial patterns, such as the dominance of *Firmicutes* and *Bacteroidota* in healthy tigers and the presence of *Proteobacteria* in tigers experiencing health stress. This indicates that microbial dysbiosis is a consistent feature across various captive tiger populations.

Our study provides valuable insights into the gut microbiome of captive Siberian tigers, highlighting the importance of personalized health and dietary management in captivity despite the limited sample size. Future research should increase the sample size, compare it with wild tiger populations, and incorporate functional analyses, such as metagenomics and metabolomics, to explore the metabolic capabilities of the gut microbiome. Longitudinal studies are also essential for tracking microbial changes over time, enabling conservationists and veterinarians to develop more effective strategies for maintaining the health and longevity of endangered tigers.

## Figures and Tables

**Table 1:** Characteristics of Siberian Tigers included in the study.

Sample ID	Age (Years)	Diet (kg/Day)	Weight (kg)	Sex
Tiger 1	11	Chicken 4–5 kg, Beef 1.5 kg	178.5	Male
Tiger 2	2	Chicken 4–5 kg, Beef 1.5 kg	178	Male
Tiger 3	9	Chicken 4–5 kg, Beef 1.5 kg	202	Male
Tiger 4	2	Chicken 3–4 kg, Beef 1.5 kg	122	Female
Tiger 5	17	Chicken 3–4 kg, Beef 1.5 kg	157.5	Female
Tiger 6	9	Chicken 4–5 kg, Beef 1.5 kg	202	Male

**Table 2:** Beta diversity analysis results for Siberian tigers.

Analysis	Comparison	Bray–Curtis Dissimilarity	Jaccard Distance	Unweighted UniFrac	Weighted UniFrac	PERMANOVA *p*-Value
1	Group A (Tigers 1, 2, and 3) vs. B (Tigers 4, 5, and 6)	0.365	0.247	0.462	0.382	0.015
2	Group A (Tigers 1, 2, and 4) vs. B (Tigers 3, 5, and 6)	0.298	0.183	0.41	0.335	0.024
3	Group A (Tigers 2, 4, and 5) vs. B (Tigers 1, 3, and 6)	0.342	0.221	0.438	0.368	0.019

**Table 3:** Dominant taxa at each taxonomic level among the six Siberian Tigers.

Taxonomic Level	Dominant Taxon	Relative Abundance (%)
Phylum	*Firmicutes*	52.8
Class	*Clostridia*	38.4
Order	*Clostridiales*	34.2
Family	*Lachnospiraceae*	27.6
Genus	*Clostridium sensu stricto*	19.5

## Data Availability

The data presented in this study are available on request from the corresponding author. The data are not publicly available due to privacy concerns regarding the individual tigers involved in the study conducted at the Korean Tiger Conservation Center.

## Supplementary Materials

The following supporting information can be downloaded at: https://www.mdpi.com/article/10.3390/microorganisms12112165/s1, Figure S1: Detailed results of the Wilcoxon Rank-Sum Test comparing alpha diversity indices between individual tigers; Figure S2: NMDS plot using Bray–Curtis dissimilarity, depicting the microbial community composition of the six tigers; Figure S3: NMDS plot using Jaccard distance, displaying the microbial community composition of the six tigers; Figure S4: NMDS plots using unweighted UniFrac and weighted UniFrac metrics, representing the phylogenetic dissimilarity of microbial communities in the six tigers; Figure S5: Stacked bar plot illustrating the relative abundance of microbial communities at the Class level among six Siberian tigers; Figure S6: Stacked bar plot showing the relative abundance of microbial communities at the Order level among six Siberian tigers; Figure S7: Stacked bar plot depicting the relative abundance of microbial communities at the Family level among six Siberian tigers.
