# Supplementary material for: Gut Microbiome Diversity and Composition in Captive Siberian Tigers (Panthera tigris altaica): The Influence of Diet, Health Status, and Captivity on Microbial Communities
Source: Microorganisms. 2024 Oct 27;12(11):2165. doi: 10.3390/microorganisms12112165 (PMC11596243; doi:10.3390/microorganisms12112165)
Supplement: Supplementary file 1 [file microorganisms-12-02165-s001.zip › microorganisms-3237269-supplementary.pdf]

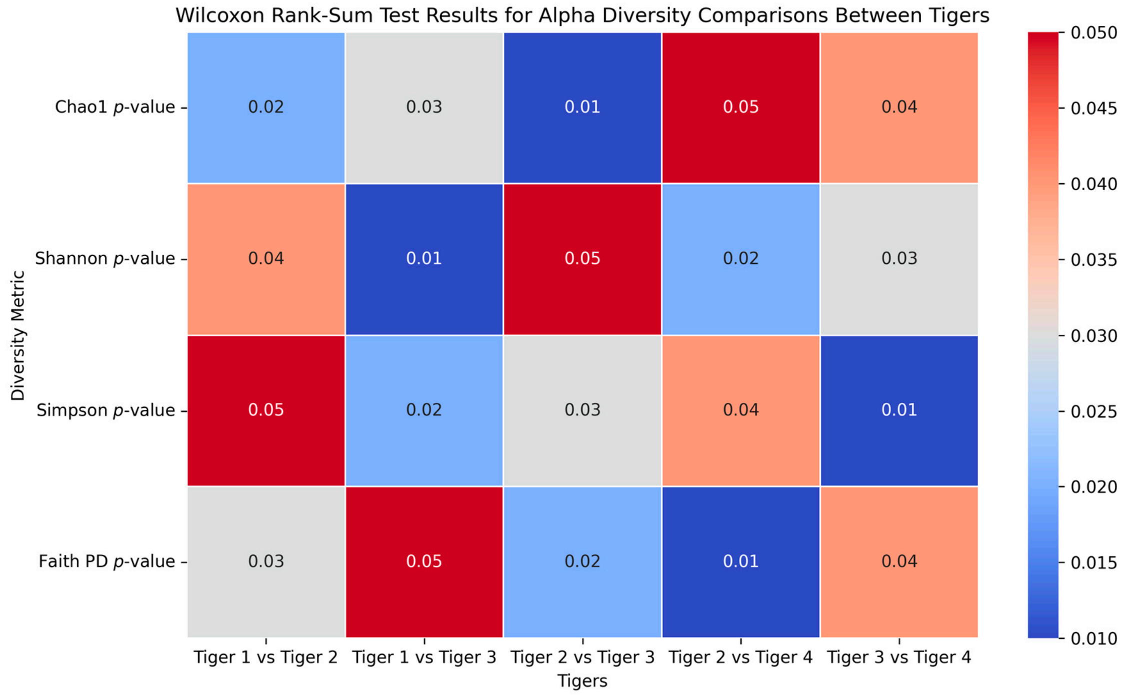

Supplementary Figure S1. Detailed results of the Wilcoxon Rank-Sum Test comparing alpha diversity indices between individual tigers.

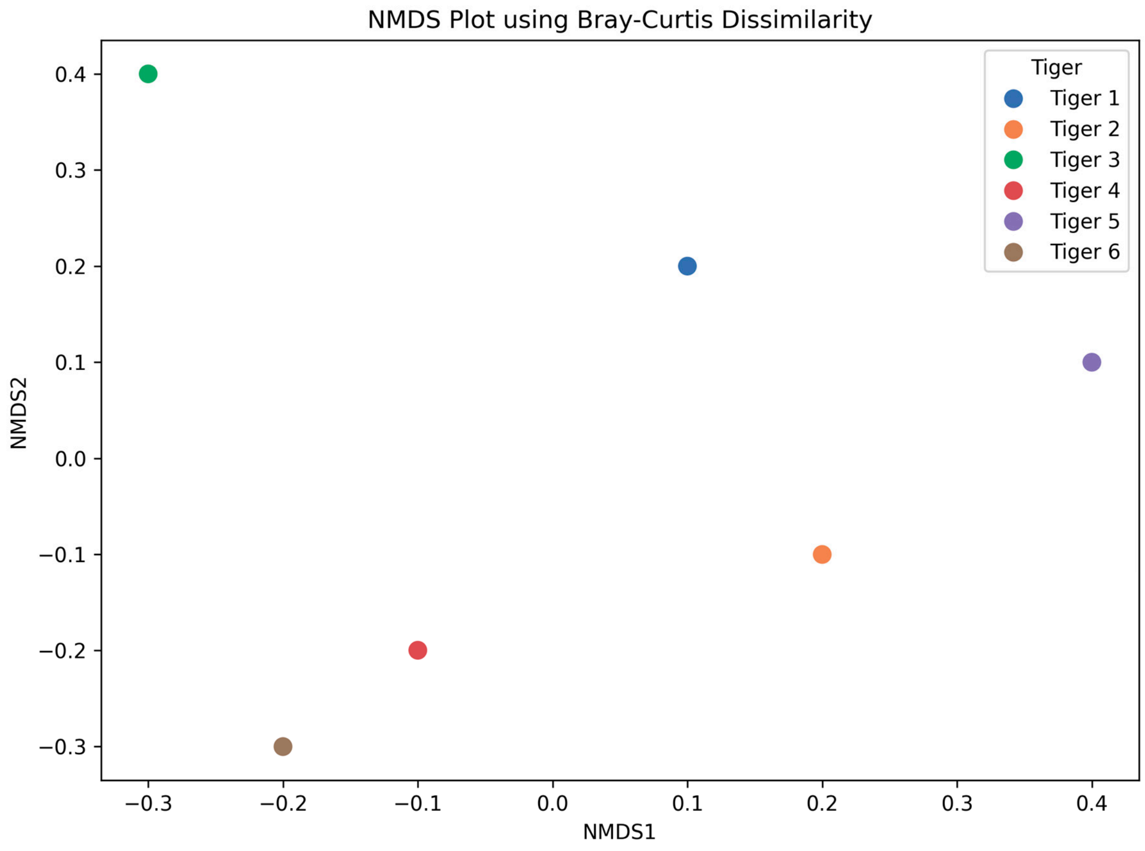

Supplementary Figure S2. NMDS plot using Bray–Curtis dissimilarity, depicting the microbial community composition of the six tigers.

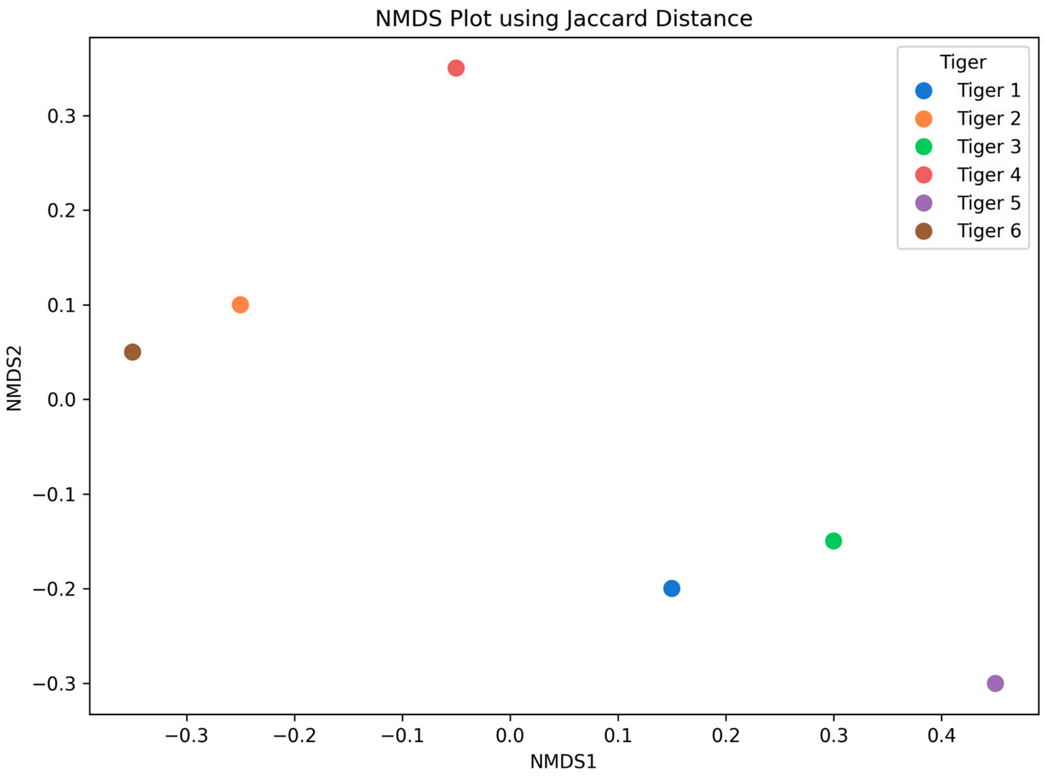

Supplementary Figure S 3. NMDS plot using Jaccard distance, displaying the microbial community composition of the six tigers.

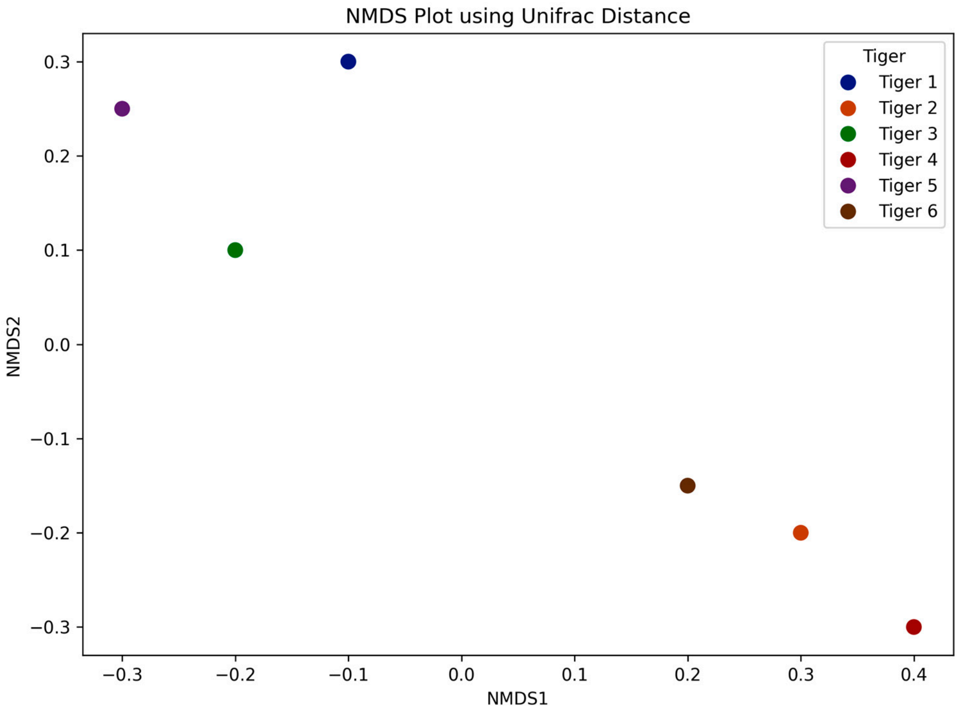

Supplementary Figure S4. NMDS plots using unweighted UniFrac and weighted UniFrac metrics, representing the phylogenetic dissimilarity of microbial communities in the six tigers.

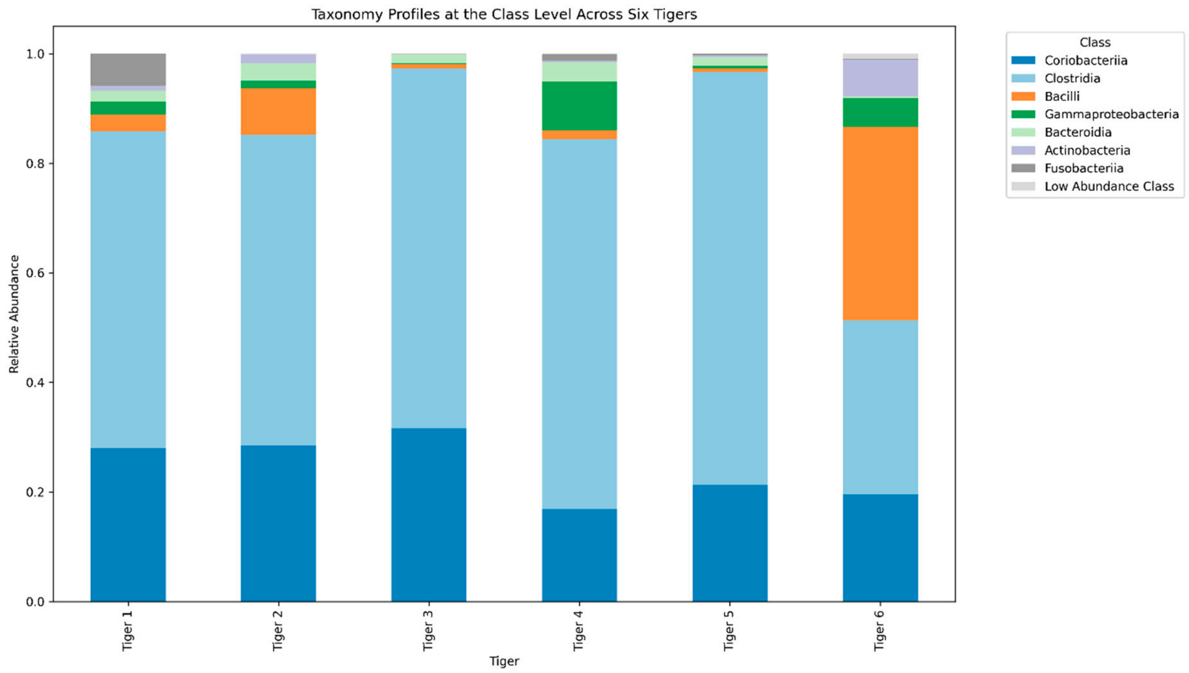

Supplementary Figure S5. Stacked bar plot illustrating the relative abundance of microbial communities at the Class level among six Siberian tigers.

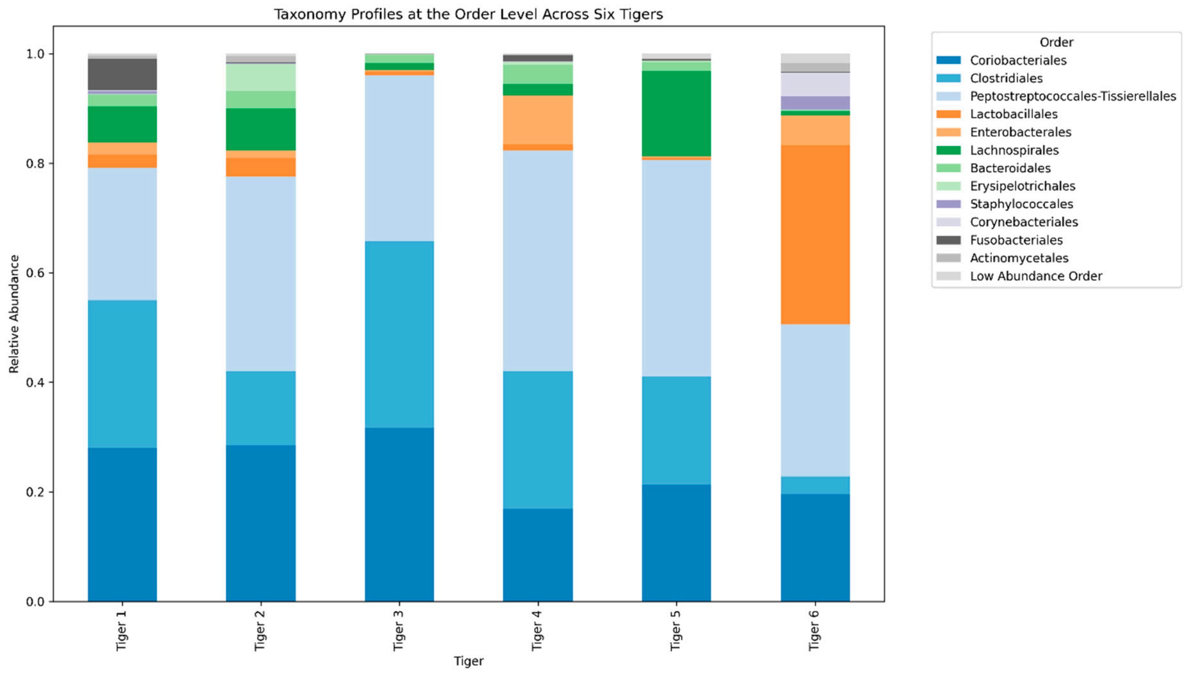

Supplementary Figure S6. Stacked bar plot showing the relative abundance of microbial communities at the Order level among six Siberian tigers.

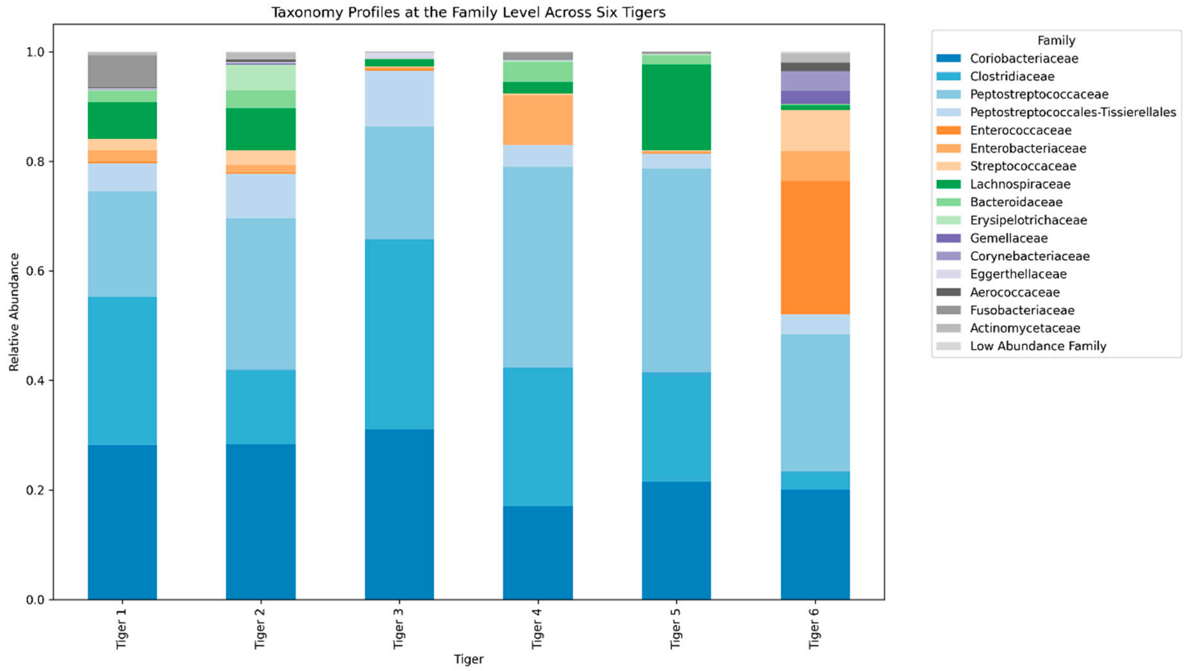

Supplementary Figure S7. Stacked bar plot depicting the relative abundance of microbial communities at the Family level among six Siberian tigers.
